# Profiles Vulnerable to Maladaptive Use of Recreational Digital Environments Identified Using the Big Five Model

**DOI:** 10.3390/bs15121749

**Published:** 2025-12-18

**Authors:** Bárbara Caffarel-Rodríguez, Andrés González Llamas, Elena Porras-García

**Affiliations:** 1 Department of Audiovisual Communication and Advertising Studies, Rey Juan Carlos University, 28942 Madrid, Spain; barbara.caffarel@urjc.es; 2 Department of Communication and Sociology, Rey Juan Carlos University, 28942 Madrid, Spain; andres.gllamas@urjc.es; 3 Department of Physiology, Anatomy and Cell Biology Pablo de Olavide University, 41013 Seville, Spain

**Keywords:** Big five, addiction, social media, cognition, neuroanatomy, digital leisure and harmful use of the internet

## Abstract

The Big Five Model has been widely applied across various areas for detecting problematic or even antisocial behaviors. This research explores its potential to identify behavior patterns and usage profiles in digital environments, such as social media use, digital gaming, and related activities. This study first conducted a literature review on mobile phone use, video game addiction, and social media overuse through the lens of the Big Five Model. Then, empirical data from 492 participants were analyzed to assess how each personality trait is associated with exposure to excessive internet use. The results shown that individuals with high openness and extraversion are more likely to engage intensively with social media and online entertainment, whereas those with higher levels of neuroticism, agreeableness, or conscientiousness display lower exposure. These findings align with previous research linking personality traits to neuroanatomical patterns that shape behavioral tendencies. This study suggests that specific personality traits, as defined by the Big Five Model, influence the use of digital media and advertising channels, potentially fostering addictive behaviors in users with higher openness and extraversion.

## 1. Introduction

### 1.1. Big Five Personality Theory

The Big Five Model originated with the lexical hypothesis (Smits & Boeck, 2006; Sánchez & Ledesma, 2007; Arterberry et al., 2014), rooted in Galton’s (1883) *Inquiry into Human Faculties and Its Development* (Ter Laak, 1996), which proposed language as the basis for studying personality. Historically, personality research has sought to explain individual behavioral differences (Montaño Sinisterra et al., 2009).

Trait theory emerged from this lexical tradition. Allport and Odbert (1936) argued that everyday language reflects personality characteristics, extending Galton’s premise that self-descriptive terms capture thoughts, behaviors, and emotions (Gónzalez Llamas, 2024). Early lexical classifications organized personality descriptors (Saucier & Srivastava, 2015), and later work led to structured personality assessments (John et al., 2008), reinforcing the construct’s relevance in psychology (Lara et al., 2021).

Raymond Cattell advanced the field by emphasizing measurable personality dimensions and developing the 16 Personality Factor Questionnaire (Cattell & Mead, in Boyle et al., 2008; Rubio, 2022). Tupes and Christal (1992) later identified five major dimensions in military studies (Ruiz, 2002). Goldberg popularized the term Big Five (Karaman et al., 2010), while Costa and McCrae formalized the contemporary model (De Raad, 1998; John et al., 2008), now widely recognized as the most empirically supported structure (Wilt & Revelle, 2015).

Despite critiques, DeYoung (2020) reinforces its cross-cultural validity, and his Cybernetic Big Five Theory (DeYoung, 2015) incorporates regulatory processes. Abood (2019) considers it the most influential framework due to its bipolar dimensions, consensus, precision, and global applicability.

### 1.2. Neuroscience of the Big Five

The Big Five is a valuable framework for defining personality traits and predicting behavioral patterns, and several neuroanatomical variations have been linked to its dimensions. Conscientiousness and openness primarily involve the prefrontal cortex, whereas extraversion and neuroticism relate to the orbitofrontal cortex (Allen & DeYoung, 2017; Restrepo, 2015).

High neuroticism is associated with greater cortical thickness and reduced gyrification in prefrontal and temporal cortices, affecting emotional regulation, impulsivity, and stress control. In contrast, openness corresponds to reduced cortical thickness but increased surface area and gyrification in prefrontal and parietal regions, supporting abstract thinking, curiosity, and novelty seeking (Allen & DeYoung, 2017; Riccelli et al., 2017; Lewis et al., 2018).

Agreeableness correlates with lower prefrontal cortical thickness and reduced temporal lobe surface area, consistent with empathetic tendencies. Extraversion has been linked to a thicker orbitofrontal cortex, a region central to reward processing and sociability. Conscientious individuals show a thicker but smaller prefrontal cortex involved in planning, decision-making, and impulse control (Lewis et al., 2018; Nostro et al., 2017; Riccelli et al., 2017).

Riccelli et al. (2017) suggest that cortical thickness and gyrification patterns—opposing between neuroticism, extraversion, conscientiousness, and openness—may reflect maturational brain effects and help explain neural predispositions to psychiatric vulnerability or resilience.

### 1.3. Social Networks and the Digital Environment

In 2012, the National Observatory of Telecommunications and the Information Society noted that, despite the variety of emerging theories, researchers converged on definitions still used today. Social networks were described as platforms for interaction, communication, content sharing, and community creation, as well as tools that democratize information by turning users into both receivers and producers (Observatorio Nacional de las Telecomunicaciones y de la Sociedad de la Información [ONTSI], 2012). This aligns with Uses and Gratifications Theory (Katz et al., 1973), which argues that individuals use media to satisfy needs such as entertainment, social relationships, identity, and surveillance—dimensions associated with Big Five traits (Martinez Gutierrez, 2010). IAB Spain (2024) further conceptualizes social networks as structures linking individuals through shared interests.

The World Health Organization defines addictions as physical and psycho-emotional dependencies involving substances or behaviors, including excessive digital use. Addictive behaviors alter brain reward systems (Blum et al., 2014) and relate to neural changes (de Sola Gutiérrez et al., 2013; Lin et al., 2012; Yuan et al., 2012). Personality predicts digital addictions: neuroticism and extraversion in mobile overuse (Takao et al., 2009; Takao, 2014), and extraversion, agreeableness, and neuroticism in shopping addiction (Mikołajczak-Degrauwe et al., 2012). Dopaminergic pathways also influence reward-driven behaviors (Becoña, 2002). Recent findings (Montag et al., 2019) show that extroverted and open individuals use social networks more, conscientious users self-regulate, agreeable users seek positive interaction, and neurotic individuals rely on digital platforms for emotional support.

With 88.9% of Spaniards over 14 using the Internet daily and 92.8% accessing it via mobile (AIMC, 2025), studying personality in digital contexts is essential.

### 1.4. The Big Five and the Prevention

Since McCrae and Costa introduced the foundations of the Big Five, its applications have expanded across multiple disciplines (De Raad, 1998; John et al., 2008; Bainbridge et al., 2022; McCrae & Costa, 2008). These include clinical and personality psychology, organizational contexts, education, and neuroscience. González Llamas (2025) highlights its preventive relevance for psychological disorders, addictions, and antisocial behaviors.

The model’s value lies in capturing stable affective, cognitive, and behavioral patterns (Wilt & Revelle, 2015), enabling objective identification of risk profiles (Cervone & Winer, 2010) and personalized prevention strategies (Pilch et al., 2021). Takao et al. (2009) show that personality predicts problematic mobile phone use, suggesting early interventions to promote self-control.

Bunz (2021) emphasizes practical implications for mental health and education, noting that personality-based patterns inform interventions in digital environments. Chittaranjan et al. (2011) found that smartphone usage aligns with personality traits, with extraversion and conscientiousness emerging as the strongest predictors. Meta-analytic evidence also links high neuroticism and low conscientiousness to problematic mobile use (Marengo et al., 2020). Cyberbullying research on Twitter/X associates aggression with high extraversion, low agreeableness, and high neuroticism (Balakrishnan et al., 2019).

Finally, Bunz (2021) reports that openness predicts greater digital use, conscientiousness relates to functional use, extraversion shows mixed results, and neuroticism reveals gender differences. Her findings indicate that social media affects subjective well-being in trait-specific ways.

### 1.5. Study Objectives

In this work, we will analyze the use of social networks based on the personality components of the Big Five model.

O1—To identify whether there is a prevalence, based on the personality traits measured with the Big Five, of addictions or inappropriate uses of the Internet.
O2—To identify whether all the traits correspond to the prevalence of addictions and misuse.
O3—To relate these personality traits and possible addictive tendencies to the neuroanatomical variations in the brain previously described in other articles.

## 2. Materials and Methods

### 2.1. Research Phases

Initially, this study was grounded in an earlier article that offered empirical research using a quite different approach. The original work by González Llamas and Ortiz de Guinea Ayala (2025), published in the Revista de Ciencias de la Comunicación e Información, analyzed exposure to a wide range of advertising channels and formats according to personality traits measured by the Big Five. While that study examined both traditional and digital advertising environments, the present research focuses specifically on digital formats that may be prone to generating addictive behaviors.

In the previous study, affinity with the digital environment was examined within a broader advertising context. Digital media were analyzed alongside television commercials, outdoor advertising, and product placement. Because the key question referred to the attention paid to these media, which presupposed prior consumer behavior, it was considered relevant to explore how these findings might contribute to identifying potential inappropriate uses of digital media.

It is important to emphasize that exposure to advertising can serve as an indicator of the degree of consumption and attention directed toward digital channels. The measured variables included Internet use through YouTube advertisements, Instagram Stories, TikTok usage, following influencers or YouTubers, Spotify usage, overall consumption of social networks, digital press consumption, Google advertising, online games, games on tablets or mobile phones, and the use of SMS or email.

In parallel, a literature review was carried out on addictions in the digital environment, the role of the Big Five in addiction research in general, and, more specifically, in digital contexts, along with a review of the psychoneurological implications associated with Big Five personality traits.

### 2.2. Empirical Sample

According to the reference article, the research initially collected 500 surveys, although 8 were excluded due to low reliability in the social desirability data. The final sample consisted of 492 participants, evenly distributed by gender (50%) and aged between 30 and 55 years.

The study was conducted through a digital panel and included two questionnaires: one measuring affinity toward brands, products, and advertising channels and formats, assessed on an eight-point scale widely used in the Spanish market; and another consisting of a version of the B.F.Q. (Spanish Big Five Questionnaire), a widely validated instrument that measures personality traits on a four-point scale. Both questionnaires were anonymized, and each participant received an identification code to unify the data. The surveys were distributed randomly across all Nielsen areas in Spain.

A Pearson correlation analysis was subsequently performed. A significance level of 0.05 or less was considered relevant, within a 95 percent confidence interval. Based on the final sample size and assuming P = Q, the margin of error for the survey was 4.31 percent.

To determine whether the differences between the items (such as YouTube ads, social media, and other variables) and the Big Five personality traits were statistically significant, an ANOVA was conducted, followed by Scheffé’s post hoc test. All analyses were performed using SPSS version 22.0 (IBM SPSS Inc., Chicago, IL, USA).

The empirical research received approval from the Research Ethics Committee of Universidad Rey Juan Carlos (URJC), with internal registration number 09012024019202.

## 3. Results

According to the results obtained, openness to experience and extraversion seem to be directly related to Internet consumption across all measured variables (see Table 1 and Table 2). When levels of openness or extraversion are high, the data show correlations with the use of different types of online content. At low levels, however, these traits do not exhibit significant correlations.

Conscientiousness appears as an intermediate trait. Although some relationships are observed, they are weaker than those found for openness and extraversion. Agreeableness, at both high and low levels, and neuroticism, mainly in low values, show only weak correlations.

These initial results suggest that the personality traits measured through the Big Five help differentiate degrees of affinity, consumption, and use of Internet and social media content.

### 3.1. Openness to Experience

Openness to experience, when present at high levels, appears to be related to the use of the Internet and social networks in all their forms. This pattern is consistent with the nature of the trait, which is typically associated with curiosity, preference for novelty, and an interest in diverse and aesthetically rich experiences. Individuals high in openness often avoid conventional environments and seek out alternative spaces, which helps explain why the Internet—an ecosystem full of varied ideas, perspectives, and interactive possibilities—fits naturally with their motivations. Their greater engagement with online games follows the same logic, as these offer stimulation, exploration, and dynamic scenarios.

Conversely, the absence of notable correlations among individuals with low openness to experience is unsurprising. This group usually reflects a more traditional, practical, and conservative profile. Although they may use the Internet when necessary, their preference tends to lean toward more familiar, stable, and straightforward media environments.

### 3.2. Extraversion

Individuals with high levels of extraversion are usually described as friendly, sociable, and energetic. Some studies suggest that they may also adopt more dominant roles in social interactions, given their tendency to express their views with greater intensity. In certain situations, this assertiveness may resemble confrontational behavior in digital environments, although this does not imply that such tendencies are widespread among all extroverted individuals.

In accordance with this profile, the data indicate a correlation between extraversion and intensive consumption of digital content, particularly social networks, digital press, and games on mobile devices or tablets. Their enthusiasm for social interaction and their high activity levels make them more likely to engage with a broad range of digital formats. The elevated scores on overall social media use also point toward a marked inclination for community building and interpersonal exchange, patterns that have been documented consistently in previous research.

### 3.3. Agreeableness

Regarding agreeableness, this trait shows associations with YouTube advertising, digital press consumption, Google advertising, and the use of SMS or email (see Table 3). Overall, the pattern observed is somewhat unexpected. The correlations appear limited to a relatively narrow set of digital media and, contrary to what might initially be assumed, agreeableness does not seem strongly aligned with social networks, online games, or other platforms that depend heavily on broad digital interaction.

Agreeable individuals are typically characterized by cooperation, empathy, and a preference for maintaining harmonious relationships. These tendencies may help explain why their engagement with digital environments appears more selective. Rather than gravitating toward highly dynamic or impersonal platforms, they may prefer communication channels that offer a more direct, familiar, or personal connection.

Taken together, the results suggest that agreeable individuals are not especially inclined toward intensive digital use. Their social orientation seems to be expressed more readily through direct, human interaction than through wider digital environments, which may account for the more limited pattern of associations observed in this trait.

### 3.4. Neuroticism

Neuroticism does not show a strong presence within the digital environment. Low levels of neuroticism display only slight but significant correlations with the consumption of YouTube advertising and digital press, which may be explained by the greater sense of control, choice, and personalization that these formats provide (see Table 4). Overall, the pattern observed suggests that neuroticism offers limited predictive value for digital consumption.

The data suggest that individuals with high levels of this trait may be active online primarily in situations involving strong emotional reactions, whether positive or negative. This is consistent with the tendency of neurotic individuals to respond more intensely to experiences that trigger frustration, validation, or emotional arousal. At the same time, given their generally insecure and distrustful disposition, it is plausible that these individuals do use digital environments, although these settings may not substantially contribute to their sense of subjective well-being.

### 3.5. Conscientiousness

Regarding the profile of conscientious individuals, this trait is generally associated with strong organizational skills, self-discipline, and a pronounced sense of responsibility. People who score high in conscientiousness tend to inspire trust in both personal and professional contexts, and they typically show high levels of self-control and self-demand, which translate into sustained commitment to the tasks they undertake.

According to the data presented in the Table 5, one of the associations appears with the use of email and SMS, a pattern consistent with the task-oriented nature of conscientious individuals. This profile also shows correlations with several other digital formats, reinforcing the idea that they tend to interact with media selectively and in a functional way. The positive associations with digital press and Google advertising may reflect an interest in informative and utilitarian content that supports their need to stay informed and work efficiently. Although to a lesser extent, small correlations were also found with general social media use and Spotify, suggesting a moderate relationship with entertainment consumption—possibly linked to moments of organization or time management rather than spontaneous or impulsive leisure.

In contrast, low scores were observed for online games and games on mobile devices. This tendency is consistent with the limited alignment between these forms of entertainment and the productive or goal-oriented habits commonly associated with conscientious individuals.

With respect to the results obtained through the ANOVA test for the different items, all differences were found to be statistically significant. The values were as follows: Youtube Ads: F(_2, 488_) = 16,537, *p* < 0,001; Stories Instagram: F(_2, 466_) = 7953, *p* < 0.001; TikTok: F(_2, 448_) = 3898, *p* < 0.05; Influencers youtubers: F(_2, 477_) = 3353, *p* < 0.05; Spotify: F(_2, 456_) = 6926, *p* < 0.001; General Social Media: F(_2, 487_) = 9809, *p* < 0.001; Digital Press: F(_2, 486_) = 14,319, *p* < 0.001; Advertising on Google: F(_2, 489_) = 14,071, *p* < 0.001; Online Games: F(_2, 470_) = 10,160, *p* < 0.001; SMS/E-mail: F(_2, 490_) = 13,143, *p* < 0.001.

Taken together, the correlation patterns and the ANOVA findings provide a coherent picture of how personality traits relate to digital media use. The correlations reveal the direction and strength of associations, while the ANOVA results confirm that these differences across personality levels are statistically robust. In practical terms, this means that digital consumption does not vary randomly but tends to differ systematically according to personality profiles. Integrating both analyses makes it possible to identify the traits most consistently linked with specific digital behaviors—particularly openness, extraversion, and conscientiousness—as well as those for which the associations are weaker, allowing for a more nuanced interpretation of individual differences in digital engagement.

## 4. Discussion

The results obtained in this study suggest that high levels of openness to experience (Table 1) and extraversion (Table 2) are significantly related to all measured variables. These findings are consistent with those reported by other authors (Bunz, 2021; Eck & Gebauer, 2022; González Llamas, 2025; Wilt & Revelle, 2015) and align with tendencies commonly associated with these traits, such as curiosity, a preference for novelty, and greater social engagement. However, the remaining personality traits appear to relate to the measured variables in distinct ways.

Agreeable profiles in our sample tended to interact more frequently with YouTube ads, digital press, Google advertising, and SMS or email (Table 3). These results are consistent with Chittaranjan et al. (2011), who suggest that agreeable individuals may prefer interactions perceived as closer or more familiar rather than those associated with more impersonal platforms like TikTok. From a neuroanatomical perspective, our findings are also consistent with those of Riccelli et al. (2017), who highlight the relevance of brain structures involved in facial recognition to agreeable behavior.

Previous research has shown differences in prefrontal cortical thickness among individuals high in conscientiousness, a structure associated with planning, impulse control, and decision-making (Lewis et al., 2018; Nostro et al., 2017; Riccelli et al., 2017). These results align with our findings and with those reported by other authors (Montag et al., 2019), suggesting that individuals scoring high on this trait may be more inclined to use email, SMS, digital press, and Google advertising. Such tendencies may reflect work-related commitment, productivity-oriented habits, and a preference for structured and reliable information sources. Regarding leisure activities, individuals high in conscientiousness appeared to prefer general social networks or Spotify over online or mobile gaming, a pattern also noted by Balakrishnan et al. (2019).

In conclusion, our study suggests that high conscientiousness may act as a protective factor against digital misuse, as proposed by Marengo et al. (2020). A similar tendency may apply to high agreeableness, perhaps due to a stronger preference for personal interaction. High neuroticism appears to play a more ambivalent role, as it may be associated with both pleasurable and stressful experiences, with the latter potentially discouraging intensive digital engagement. High openness to experience was linked to a broad range of digital options, as also noted by Bunz (2021). This may be related to these individuals’ inclination to explore new stimuli and acquire new knowledge; however, such exploratory tendencies could contribute to more dispersed patterns of engagement, which might explain why clear addiction profiles are not consistently identified despite strong affinities.

Finally, high extraversion, in addition to showing a wide array of preferences across digital formats, appears to be among the traits most closely associated with vulnerability to excessive use of social networks, digital games, online leisure, and the Internet more generally. This is a highly energetic and socially oriented group. Montag et al. (2019) note that these individuals tend to have a strong need for communication, and Balakrishnan et al. (2019) observe that they may display a higher prevalence of behaviors resembling bullying tendencies as a means of enhancing their self-perceived status. In this sense, intensive engagement with digital platforms may be linked to the self-esteem reinforcement derived from such activity. Since social networks and digital games provide environments characterized by interaction and self-expression—needs that are particularly salient for extroverts—they may facilitate the emergence of potentially problematic patterns of use.

When performing this study, we encountered several limitations that should be considered when interpreting the results. First, the sample size did not allow for a breakdown by age groups, so the findings correspond to an adult population aged 30 to 55. The same limitation applies to gender, which could not be analyzed in depth. Second, certain confounding variables may have introduced bias, such as the underlying motivations for using the Internet—whether for leisure, work, or other purposes—which were not controlled for in this design.

Regarding implications, the findings highlight the relevance of the digital environment and confirm that personality traits do play a role in shaping online behaviors and media consumption patterns. However, the present research approached personality from a single-trait perspective, and personality results from the interaction of all five traits. Future lines of research could benefit from incorporating statistical models that examine how combinations of traits jointly influence digital behavior, allowing for a more comprehensive understanding of prevalence patterns associated with the digital ecosystem.

In summary, examining digital consumption through the lens of the Big Five framework makes it possible to identify individuals who may be more likely to develop problematic or compulsive patterns of online behavior. In this study, two traits appear particularly relevant in shaping these tendencies. Extraversion, with its strong orientation toward communication and social engagement, and openness to experience, which encourages exploration and the search for novel experiences, both seem to be associated with more intensive and occasionally more fragmented or conflictual patterns of digital use. This is especially evident among individuals who score high in extraversion.

Conversely, high conscientiousness appears to function as a protective factor against maladaptive or compulsive digital use, as this trait promotes order, self-regulation, and a more rational approach to media consumption. Similarly, agreeableness tends to favor direct, face-to-face interaction, which may explain why, although individuals high in this trait do engage with digital media, the trait itself does not strongly predict intensive or compulsive online behavior, and even less so behaviors that may be conflictual.

Finally, neuroticism, as observed in much of the existing literature, does not seem to be a consistent predictor of general digital media consumption within this study, although its influence may emerge in more emotion-driven contexts.

## Figures and Tables

**Table 1:** Correlation between the personality trait “Openness to Experience” and the consumption of advertising/social networks.

	Openness to Experience		
	Pearson Correlation	Sig. (Bilateral)	N
Youtube Ads	0.249 **	0.001	491
Stories Instagram	0.201 **	0.001	469
TikTok	0.133 **	0.005	451
Influencers youtubers	0.143 **	0.002	480
Spotify	0.186 **	0.001	459
General Social Networks (SNS)	0.217 **	0.001	490
Digital Press	0.248 **	0.001	489
Advertising on Google	0.241 **	0.001	492
Online Games	0.210 **	0.001	473
Tablet/Mobile Games	0.176 **	0.001	474
SMS/E-mail	0.225 **	0.001	493

Abbreviations: Sig: significance; ** *p* < 0.01.

**Table 2:** Correlation between the personality trait “Extraversion” and the consumption of advertising/social networks.

	Extroversion		
	Pearson Correlation	Sig. (Bilateral)	N
Youtube Ads	0.208 **	0.001	491
Stories Instagram	0.193 **	0.001	469
TikTok	0.197 **	0.001	451
Influencers youtubers	0.165 **	0.001	480
Spotify	0.183 **	0.001	459
General Social Networks (SNS)	0.186 **	0.001	490
Digital Press	0.232 **	0.001	489
Advertising on Google	0.218 **	0.001	492
Online Games	0.193 **	0.001	473
Tablet/Mobile Games	0.169 **	0.001	474
SMS/E-mail	0.216 **	0.001	493

Abbreviations: Sig: significance; ** *p* < 0.01.

**Table 3:** Correlation between the personality trait “Agreeableness” and the consumption of advertising/social networks.

	Agreeableness		
	Pearson Correlation	Sig. (Bilateral)	N
Youtube Ads	0.124 **	0.006	491
Stories Instagram	0.086	0.062	469
TikTok	0.049	0.298	451
Influencers youtubers	0.011	0.814	480
Spotify	0.070	0.137	459
General Social Networks (SNS)	0.079	0.082	490
Digital Press	0.126 **	0.005	489
Advertising on Google	0.106 *	0.019	492
Online Games	0.050	0.281	473
Tablet/Mobile Games	0.052	0.261	474
SMS/E-mail	0.141 **	0.002	493

Abbreviations: Sig: significance; * *p* < 0.05, ** *p* < 0.01.

**Table 4:** Correlation between the personality trait “Neuroticism” and the consumption of advertising/social networks.

	Neuroticism		
	Pearson Correlation	Sig. (Bilateral)	N
Youtube Ads	−0.112 *	0.013	491
Stories Instagram	−0.063	0.174	469
TikTok	0.030	0.528	451
Influencers youtubers	0.023	0.614	480
Spotify	−0.025	0.591	459
General Social Media	−0.046	0.305	490
Digital Press	−0.091 *	0.044	489
Advertising on Google	−0.068	0.130	492
Online Games	−0.010	0.832	473
Tablet/Mobile Games	−0.074	0.109	474
SMS/E-mail	−0.060	0.182	493

Abbreviations: Sig: significance; * *p* < 0.05.

**Table 5:** Correlation between the personality trait “Conscientiousness” and the consumption of advertising/social networks.

	Conscientiousness		
	Pearson Correlation	Sig. (Bilateral)	N
Youtube Ads	0.184 **	0.001	491
Stories Instagram	0.110 *	0.018	469
TikTok	0.047	0.316	451
Influencers youtubers	0.017	0.716	480
Spotify	0.104 *	0.026	459
General Social Media	0.091 *	0.044	490
Digital Press	0.181 **	0.001	489
Advertising on Google	0.153 **	0.001	492
Online Games	0.056	0.228	473
Tablet/Mobile Games	0.062	0.181	474
SMS/E-mail	0.205 **	0.001	493

Abbreviations: Sig: significance; * *p* < 0.05; ** *p* < 0.01.

## Data Availability

The data presented in this study are available on request from the corresponding author due to privacy restrictions.
